# Human-AI Interaction With AI-Assisted Tumor Overlays in Pediatric Whole-Body Magnetic Resonance Imaging: Exploratory Reader Study

**DOI:** 10.2196/81066

**Published:** 2026-08-07

**Authors:** Abhishek Moturu, Olurotimi Komolafe, Sayali Joshi, Andrea S Doria, Anna Goldenberg

**Affiliations:** 1 Department of Computer Science, University of Toronto, 40 St George Street, Toronto, ON, M5S 2E4, Canada; 2 Vector Institute, Toronto, ON, Canada; 3 Genetics and Genome Biology, Hospital for Sick Children, Toronto, ON, Canada; 4 Temerty Centre for Artificial Intelligence Research and Education in Medicine, University of Toronto, Toronto, ON, Canada; 5 Department of Diagnostic & Interventional Radiology, Hospital for Sick Children, Toronto, ON, Canada; 6 Department of Medical Imaging, University of Toronto, Toronto, ON, Canada; 7 CIFAR, Toronto, ON, Canada; 8 Department of Laboratory Medicine and Pathobiology, University of Toronto, Toronto, ON, Canada

**Keywords:** machine learning, radiology, segmentation, pediatric oncology, whole-body magnetic resonance imaging, wbMRI, Li-Fraumeni syndrome, tumor detection, human-AI interaction, interpretability in AI

## Abstract

**Background:**

AI tools have the potential to enhance personalized clinical care, particularly in radiology. However, their integration into clinical workflows remains complex, especially in pediatric oncology, where early cancer detection is critical. Children with Li-Fraumeni syndrome (LFS), a rare cancer predisposition disorder, undergo regular surveillance whole-body magnetic resonance imaging (wbMRI), which presents an opportunity for AI-assisted tumor detection.

**Objective:**

We evaluated the feasibility of an AI-assisted overlay for highlighting tumor-like regions in pediatric surveillance wbMRI and explored how access to the overlay influenced radiologist workflow, candidate-lesion marking behavior, follow-up recommendations, and perceived workload.

**Methods:**

We developed a patch-based AI segmentation model trained on augmented 2D slices from 675 surveillance wbMRI volumes of pediatric patients with LFS. The model was designed to highlight regions with high tumor probability. A reader study was conducted with 2 radiologists who independently reviewed wbMRI cases both with and without AI assistance. We measured evaluation time, number and location of reader-marked candidate lesions, type of follow-up recommendation, and subjective feedback using structured questionnaires.

**Results:**

AI assistance altered interpretation workflows for both radiologists, with mixed effects. On average, the time required to evaluate each case increased when using the AI tool for both radiologists. However, one radiologist had an increase in the number of candidate lesion locations selected with the tool, and one had a decrease in the number of candidate lesion locations selected with the tool. Subjective feedback indicated that one of the radiologists reported lower mental demand with the AI tool, while both radiologists reported lower stress with the AI tool. Interrater variability was evident, underscoring the need for personalized calibration of AI tools.

**Conclusions:**

AI-assisted wbMRI interpretation can improve tumor detection in pediatric cancer surveillance by reducing false negatives. However, its influence on workflow efficiency and interradiologist variability highlights the importance of careful implementation. Successful integration requires addressing challenges such as improving the predictive precision of AI models, offering intuitive end-user designs and instructions, and building trust in AI outputs. AI outputs can influence workflow and behavior in reader-specific ways. Clinical translation will require larger, randomized, multireader studies and model refinement to reduce false positives and quantify lesion-level reader performance. This can help ensure better patient outcomes in addition to reduced clinician burnout.

## Introduction

Integrating AI tools into clinical practice marks a paradigm shift in health care, with the potential for enhanced diagnostic accuracy, improved workflows, and personalized patient care. In fact, a 2019 survey of health professionals found that up to 71% of surveyed physicians opined that AI would improve and impact the field of medicine within the next decade [1]. The potential of AI tools in health care extends across various domains, including radiology [2], pathology [3], dermatology [4], ophthalmology [5], general practice [6], surgery [7], neurology [8], cardiology [9], and more. By leveraging vast amounts of medical data, AI algorithms can help identify patterns and correlations that may be more difficult for human clinicians to recognize due to the size, rarity, or complexity of certain clinical conditions. As a result, this can enable earlier and more accurate diagnoses and subsequent interventions. Machine learning models have shown promising task-specific performance in selected medical-imaging applications, although performance and clinical benefit vary substantially across tasks, datasets, and implementation settings [10].

In addition to aiding in clinical diagnostics, AI tools can enhance clinical decision-making [11]. AI-based decision support systems can synthesize plans from multimodal data and can continuously learn from new data, improving recommendations over time and adapting to the latest clinical guidelines and research findings [12]. The use of AI in clinical settings also addresses the growing concern of clinician burnout caused by an increase in workload in interpreting imaging examinations without a corresponding increase in manpower [13]. Yu et al [14] further support the complexity of human-AI collaboration in diagnostic settings and indicate significant variability in how radiologists respond to AI assistance. They found that radiologists’ performance was easily influenced by AI errors in their large-scale study on AI-aided chest X-ray interpretation, but conventional factors such as experience or familiarity with AI did not predict the impact of AI support. There is also inherent variability in how radiologists use and interact with AI tools, which is influenced by personal biases, cognitive strategies, and the opacity of AI algorithms [15-18].

Integrating AI into clinical practice also raises concerns about data privacy [19], algorithmic bias [20], and the need for rigorous validation and regulation [21]. Ensuring that AI tools are reliable, explainable, and complement rather than replace clinical judgment is critical to ensuring their acceptance, since replacement is a significant fear in the field, for clinicians and patients receiving care alike [22,23]. Thus, a collaborative approach, where AI augments the clinician’s expertise instead of acting as a substitution, is essential for the successful adoption of these technologies.

In this paper, we studied the interaction between radiologists and a specific AI tool that was developed for the interpretation of tumors in whole-body magnetic resonance imaging (wbMRI) performed as part of an imaging protocol for cancer surveillance [24]. Early detection of tumors in patients with cancer predisposition disorders such as Li-Fraumeni syndrome (LFS) through clinical-imaging protocols has been shown to improve the prognosis of patients [25]. However, the detection and segmentation of tumors in pediatric wbMRIs is challenging for many reasons. In particular, variations in the age, sex, body shape, body size, and bone density of a developing child may influence the anatomical and physiological properties that contribute to image formation. In addition, variations in tumor size, location, magnetic resonance imaging (MRI) signal brightness, type and settings of the MRI scanner, and MRI artifacts are a few of the factors that radiologists must consider when evaluating wbMRIs in search of tumors in patients with LFS [26]. Although the malignancy of a tumor cannot always be identified from an MRI examination alone, developing a tool to assist radiologists in finding tumors by highlighting areas of high tumor probability may save time, reduce the chance of missing tumors, and minimize the amount of invasive tests. However, there is a lack of tools for finding tumors in wbMRIs for the reasons outlined above. To our knowledge, AI-assisted tumor detection has been less studied in pediatric surveillance wbMRI than in adult or single-organ imaging settings. This setting is distinct because lesions are often very small relative to the full image volume, tumor prevalence is low, and the whole-body field of view introduces substantial anatomical heterogeneity. Accordingly, our contribution is both technical and human-factors-oriented: we describe a patch-based tumor-likelihood overlay for pediatric LFS surveillance wbMRI and an exploratory reader study, evaluating how the AI overlay influences workflow, candidate-lesion marking behavior, follow-up recommendations, and perceived workload.

In order to find areas of high tumor probability within wbMRIs, we use segmentation. The use of deep learning methods to perform biomedical segmentation tasks effectively, especially in low-data availability domains, has seen great strides in recent years [27,28]. In spite of this, the comparative sizes of wbMRI volumes and tumors are so vastly different that the signal-to-noise ratios are often too low for proper tumor detection and segmentation when using full volumes or generative approaches [29,30]. Patch-based approaches show promise in this regard for segmenting very small objects in large spaces [31-33].

After developing our AI tool to highlight areas of high tumor probability in wbMRIs, we performed a validation study with 2 radiologists, each reviewing several wbMRIs with and without the assistance of the AI tool, to analyze the effect of the tool on the overall evaluation time, candidate-lesion marking behavior, follow-up recommendations, and questionnaire responses. Based on our findings from this study and relevant literature, we provide insights on how to effectively incorporate AI tools into health care to navigate existing challenges and uncover unexplored opportunities. Based on these findings and relevant literature, we discuss practical considerations for integrating such overlays into health care workflows.

## Methods

### AI Tool Development

**Figure 1.**
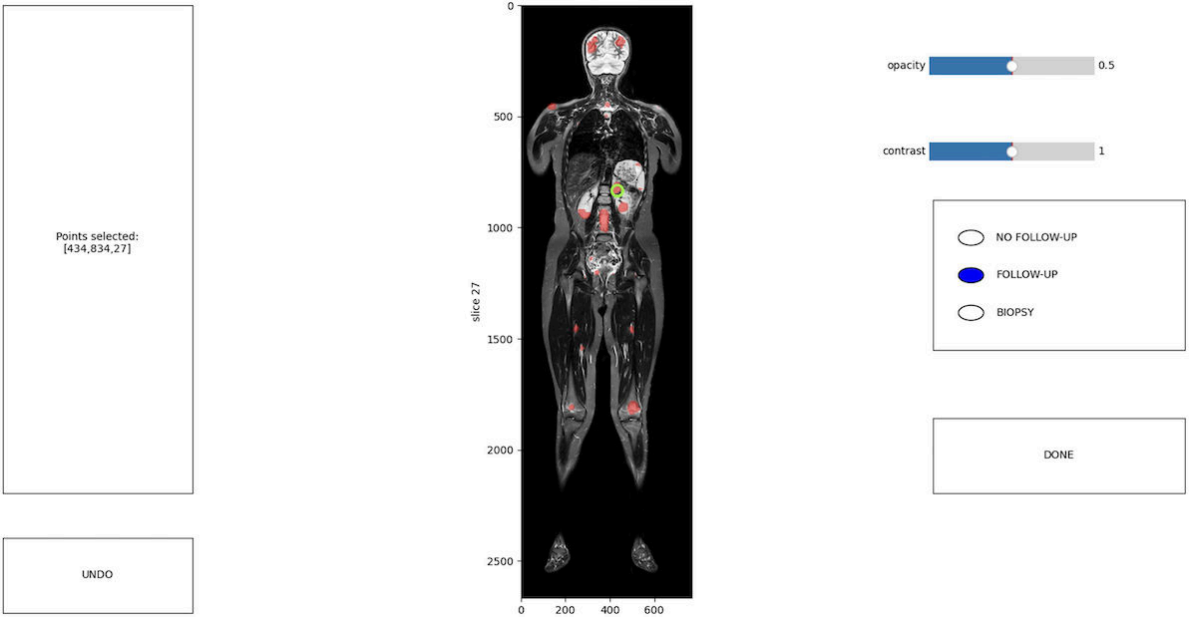
The app that allows scrolling through a volume with the AI tool overlaying masks onto the regions of high tumor probability (in red), selected points (candidate lesion locations) on the image (in a green circle), a list of selected points on the left, an opacity slider and contrast slider on the top right, and the a reader-selectable follow-up recommendation on the right.

We developed our AI tool, shown in Figure 1, starting from its initial conception and progressing through multiple iterations to refine it based on best practices and feedback. We carefully integrated feedback from our clinical collaborators at every stage, ensuring that both the underlying algorithm and the user-facing components aligned with real-world diagnostic needs. The model-development and reader-study components are reported separately below.

We trained the AI tool to recognize areas of high tumor probability using datasets generated by obtaining 2D patches from the slices of 675 coronal Short Tau Inversion Recovery (STIR) wbMRI examinations in which volumes were assessed from patients with LFS aged 0 to 18 years, followed in the Oncology Department of a large tertiary pediatric hospital after the study received Research Ethics Board approval from our institution. Only 27 out of 675 (4.0%) volumes were tumor-positive, containing 60 tumors in total, and only 120 out of 27,437 (0.44%) slices contained tumor signal. We have approximately 40 slices per volume, with each slice being approximately 3000×800 pixels. This created an extreme class-imbalance setting, with tumor tissue representing a minute fraction of the total image area. Because the natural prevalence of tumor-containing tissue was too low for stable patch-based training, patch sampling was intentionally enriched for tumor-containing patches, as described below. The diameter of each tumor section ranged from about 10 to 100 pixels, representing only a small fraction of each wbMRI volume. We used 4-fold cross-validation and a test set while ensuring that each of the train and validation folds, as well as the test set, was separated by patient volumes. A total of 5707 patches of size 128×128 pixels were extracted after split assignment, with 3995 (approximately 70%) used for training/validation and 1712 (approximately 30%) for testing. Within each split, approximately 25% (999/3995 and 428/1712) of the sampled patches contained tumor tissue. This proportion was enforced intentionally for model development and did not reflect the natural prevalence of tumor-containing tissue in surveillance wbMRI. Positive patches were drawn primarily from tumor-containing slices and centered on annotated lesions, with additional jittered samples to vary lesion position; negative patches were sampled from tumor-free regions, tumor-free volumes, and physiologically bright STIR regions that commonly generate false positives. Data splitting was performed at the patient level before patch extraction so that all patches from a given patient remained within a single training, validation, or test partition. The results on the volume-level cross-validation set and test set were obtained by running the trained patch-based model in a sliding-window manner on each slice, with 25% pixel overlap between adjacent patches to reduce edge misses.

### Ethical Considerations

The study received approval from the Research Ethics Board at The Hospital for Sick Children (SickKids) under the protocol “Machine Learning (ML) Approach to Improve Early Cancer Detection” (number 1000063239; principal investigator: AG).

### Patches, Augmentations, and Synthetic Tumors

**Figure 2.**
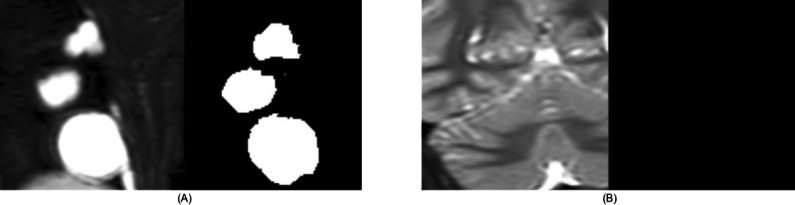
(A) A patch and mask with tumors. (B) A patch and mask without tumors.

Rather than using all possible patches, we found it more efficient and effective to primarily train the model using patches that contained tumors centrally (Figure 2). The masks for the corresponding patches were taken from the same location within the mask volumes. We also included a few patches with their centers moved around randomly to account for variations in the location of a tumor within any given patch. To improve the specificity of the tool, we also included several patches from elsewhere in the body to train the model to recognize false positives. For instance, we included patches from parts of the body that are inherently bright on wbMRI STIR sequences, to ensure that the model can differentiate between inherently bright body regions and tumors, which also appear bright. Finally, we included a few patches from volumes without tumors, which were obtained from the same general region as volumes containing tumors, in order to help the model differentiate between patches with and without tumors obtained from the same part of the body.

To improve robustness, we applied augmentations intended to approximate routine manipulation during MRI review, including zoom, brightness variation, and noise modulation. We also included synthetically inserted tumors of varying size, shape, brightness, and location to increase exposure to rare small-lesion patterns [34,35].

### Model and Losses

The U-Net model [28] was used along with a compound loss function containing asymmetric unified focal loss [36], Dice loss [37], contour perimeter loss [38], and contour difference loss to address extreme foreground-background imbalance and to improve lesion boundary localization. The respective coefficients α, β, γ, and δ of each of these losses were tunable hyperparameters.


(1) $${Loss}_{\text{patch}}=\alpha \times {Loss}_{AUF}+\beta \times {Loss}_{D}+\gamma \times {Loss}_{CP}+\delta \times {Loss}_{CD}$$

Each of the 4 parts of the loss function (Equation 1) served a specific purpose. Loss_AUF_ and Loss*_D_* dealt with the imbalance between the hard class (tumor pixels) and easy class (nontumor pixels), and Loss_CP_ and Loss_CD_ ensured that the tumor was properly localized and sized with limited overguessing or underguessing.

### Radiologist Evaluation

#### Setup

To evaluate our AI tool designed to highlight areas of high tumor probability on pediatric wbMRIs from children with a known cancer predisposition syndrome, based on the patch-based model run across wbMRIs in a sliding window fashion as described above, we gave 2 radiologists (Olurotimi Komolafe and Sayali Joshi, each with 3 years of radiology experience after training, respectively, who were supervised by a senior radiologist, Andrea S Doria, with over 20 years of radiology experience after training) detailed instructions on how to label 25 wbMRI volumes with the aid of the AI tool and 25 wbMRIs without the aid of the AI tool. The tool provided radiologists with the option to have AI overlays of predicted masks of high tumor probability regions to potentially help them identify tumors more efficiently and accurately. This was an exploratory, nonrandomized, noncounterbalanced reader study. Each radiologist reviewed 25 cases with AI overlays, followed by 25 different cases without AI overlays.

A timer was incorporated into each wbMRI, which automatically started as soon as each radiologist began the evaluation. The first 25 wbMRIs assigned to each radiologist contained the AI-assisted tumor prediction overlays. The app, as pictured in Figure 1 (note that the red prediction mask overlay is only shown for volumes 1‐25 and not shown for volumes 26‐50), shows the user interface we designed for evaluation. When reading the AI-assisted MRI volumes, the radiologists had the option to scroll up and down through each volume using the up and down keys on the keyboard (or scroll using the mouse or trackpad). They also had the option to change the degree of opacity of the red prediction mask overlays using an opacity scroller, which enabled them to transition between fully transparent and fully opaque. The default opacity was set to 50%. The app also incorporated a contrast tool to alter the visual contrast of the original wbMRI from 0 to 2, where 0=low contrast and 2=high contrast. The default contrast was set to 1. To improve detail, the app also incorporated zoom in, zoom out, and pan features, whose buttons were placed on the bottom left of the screen (Figure 1). These augmentations, some of which were suggested by the participating radiologists, were incorporated to simulate the native MRI reporting tools, which the radiologists were accustomed to in reporting wbMRIs.

The primary task was to place a click at the center of each suspected lesion or other candidate abnormality within a wbMRI volume. We refer to these clicks as candidate lesion locations rather than tumors because each click represented a reader’s suspicion rather than a verified lesion. Clicking on a region placed a green circle over that region, with a corresponding coordinate from the region added to a “Points selected” box (Figure 1). This box contained all the points (candidate lesion locations) selected for that specific wbMRI volume and served the purpose of providing visual feedback of all candidate lesion locations to the radiologists. This way, they could see if any points had been selected in error. To undo any errors, an “Undo” button was provided, which deleted the previous selection circle and the corresponding error coordinates from the selection box.

After selecting all the candidate lesion locations that they suspected to contain tumors/lesions, they were then asked to choose whether the lesion required “No Follow-Up,” “Follow-Up,” or “Biopsy.” The options to do this and finish the evaluation were provided on the right side of the screen (Figure 1). Invariably, the radiologists’ choice as to whether or not a lesion required follow-up was reflective of their consideration of the lesion to be malignant, suspicious, indeterminate (biopsy or follow-up), or benign/physiological (no follow-up). After clicking on a choice of follow-up, the incorporated timer automatically stopped, and the radiologist was redirected to a short Likert survey to evaluate their reporting experience for that particular volume. The survey evaluations were not timed.

#### Survey

The survey consisted of the following questions, ranked on a scale from 1 (“not at all”) to 5 (“a great deal”):

Q1. How mentally demanding was the task?

Q2. How hurried or rushed was the pace of the task?

Q3. How successful were you in accomplishing what you were asked to do?

Q4. How hard did you have to work to accomplish your level of performance?

Q5. How insecure, discouraged, irritated, stressed, and annoyed were you?

After concluding their reading of the first 25 wbMRI volumes, which contained tumor prediction overlays, the radiologists then read another set of 25 wbMRI volumes, following the exact same procedures, but without tumor prediction overlays. Data obtained from each MRI volume—with and without the AI overlays—included the amount of time it took to finish the labeling, the candidate lesion locations selected, the follow-up recommendation, and the survey responses.

## Results

To better study the discrepancy between the performance at the patch level and the volume level, the following metrics were considered at each level. Although it is desirable for diagnostic tools to have both low false-positive and false-negative rates, there is often a trade-off in trying to minimize both.

Given the priority of minimizing false negatives, we used a threshold to check whether at least 10% of the tumor pixels have been predicted by the segmentation model. The dice score was easily affected by the amount of tumor pixels correctly predicted and therefore did not need to be rigorously optimized, as predicting the general tumor location is sufficient. Similarly, the pixel-based false-positive rate needs to be higher for the segmentation model to properly identify parts of tumors that would otherwise be missed. We achieved a 1.54% increase on average across the validation folds in the binary tumor detection score at the patch level, as shown in Table 1, with 0.9550 using U-Net without any modifications and 0.9704 using U-Net with all of the modifications discussed in this paper.

**Table 1.** Train/validation results for each additional modification to the U-Net model with the average dice score, true-positive rate (TPR), false-positive rate (FPR), and binary tumor detection score (true for a patch if TPR>10%) across the folds^a^.

U-Net modifications	Dice score	TPR	FPR	TPR>10%
Loss*_D_*	0.9097/0.8200^b^	0.9874/0.9391	0.0080/0.0096^b^	0.9926/0.9550
+ Loss_AUF_	0.8105/0.7463	0.9796/0.9478	0.0170/0.0181	0.9911/0.9616
+ Loss_CP_	0.8182/0.7439	0.9788/0.9486	0.0167/0.0440	0.9883/0.9666
+ Loss_CD_	0.8328/0.7644	0.9834/0.9457	0.0179/0.0139	0.9908/0.9651
+ Augmentations	0.7703/0.7732	0.9767/0.9476	0.0229/0.0137	0.9865/0.9660
+ Synthetic tumors	0.8014/0.7577	0.9754/0.9527^b^	0.0167/0.0155	0.9858/0.9704^b^

a We treat true-negative patches as perfect matches, which count toward the patches where TPR >10%. Each row consists of all the additions in the previous rows.

b We denote the best validation results for each metric. Note that the best TPR and TPR>10% come from including all of the U-Net modifications.

**Figure 3.**
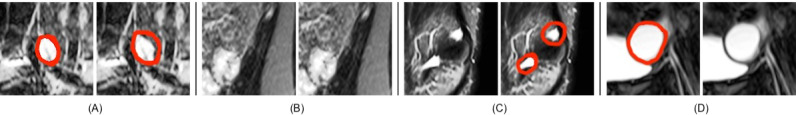
Examples of contours of the true and predicted masks (red outline) for (A) true positive, (B) true negative, (C) false positive, and (D) false negative when identifying a tumor.

**Figure 4.**
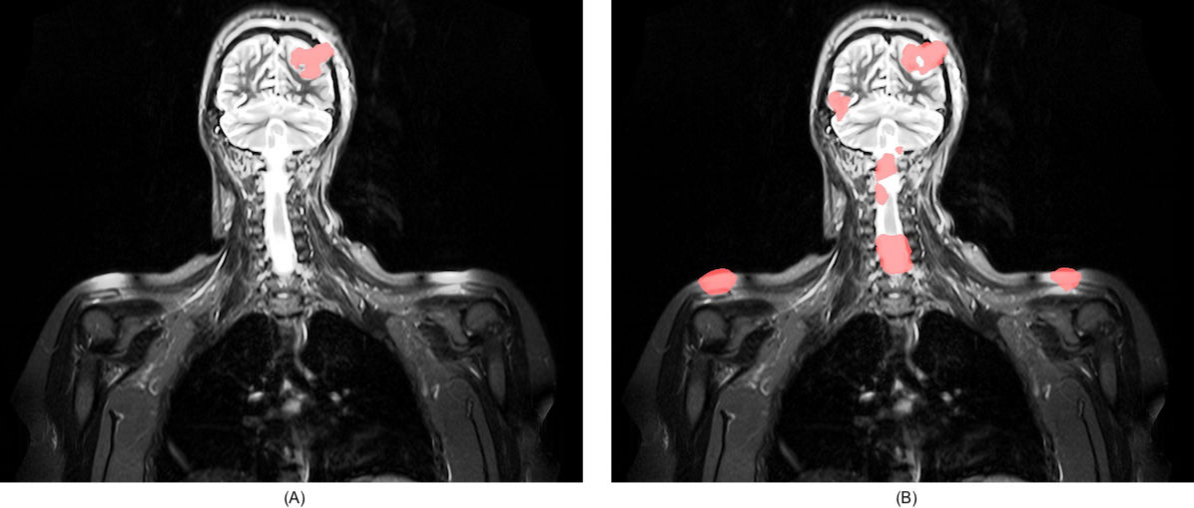
Representative test-set example. (A) Ground-truth tumor annotation. (B) Model output overlaid in red. In addition to the true lesion, nontumor highlighted areas are visible, illustrating the false-positive burden that radiologists would need to dismiss in clinical use.

At the patch level, in addition to recording each of the losses and the total weighted loss, the true-positive rate (nonthresholded and thresholded at intervals of every 10% of tumor detection), the true-negative rate, false-positive rate, false-negative rate, positive predictive value, negative predictive value, accuracy, balanced accuracy, Jaccard index, false discovery rate, and false omission rate were recorded for both the training and validation sets. These epoch-based metrics were considered when selecting the best model. Figures 3 and 4 illustrate representative test-set predictions.

The detection rates reported below are lesion-level and tumor-positive volume-level metrics from sliding-window inference on full wbMRI volumes; a tumor was counted as detected when the model overlapped at least 10% of the annotated tumor pixels. On the cross-validation set, 27 out of 53 (50.9%; 95% Wilson CI 37.9%‐63.9%) tumors were detected in 16 out of 21 (76.2%; 95% Wilson CI 54.9%‐89.4%) tumor-positive volumes. On the held-out test set, 6 out of 7 (85.7%; 95% Wilson CI 48.7%‐97.4%) tumors were detected in 5 out of 6 (83.3%; 95% Wilson CI 43.6%‐97.0%) tumor-positive volumes. Given the very small number of positive cases, these intervals are wide and should be interpreted cautiously. The false positive rates in each volume ranged from approximately 0.5% to 2%. However, it is important to note that tumors only comprised around 0.01% to 0.0001% of each given volume. Therefore, there were 50 to 20,000 times more false positive pixels (usually brighter spots not representing tumors) than true positive pixels (representing tumors). This discrepancy between the false positive vs true positive pixels may not be an issue if radiologists are experienced and can readily dismiss clear cases of groupings of false positive pixels (such as variants of normality in bone marrow signal intensity) as false positives based on their specialized knowledge and complementary clinical and laboratory assessments. This false positive burden is an important limitation because even visually dismissible false positive regions may increase reader verification workload and influence how radiologists respond to the AI overlay.

Two radiologists, designated as radiologist 1 and radiologist 2, independently assessed pediatric wbMRIs under 2 conditions: with and without the AI-assisted diagnostic tool. We systematically analyzed their differences in labeling strategies, assessment duration, recommendations for clinical follow-up, and subjective evaluations of their workflow experience.

**Figure 5.**
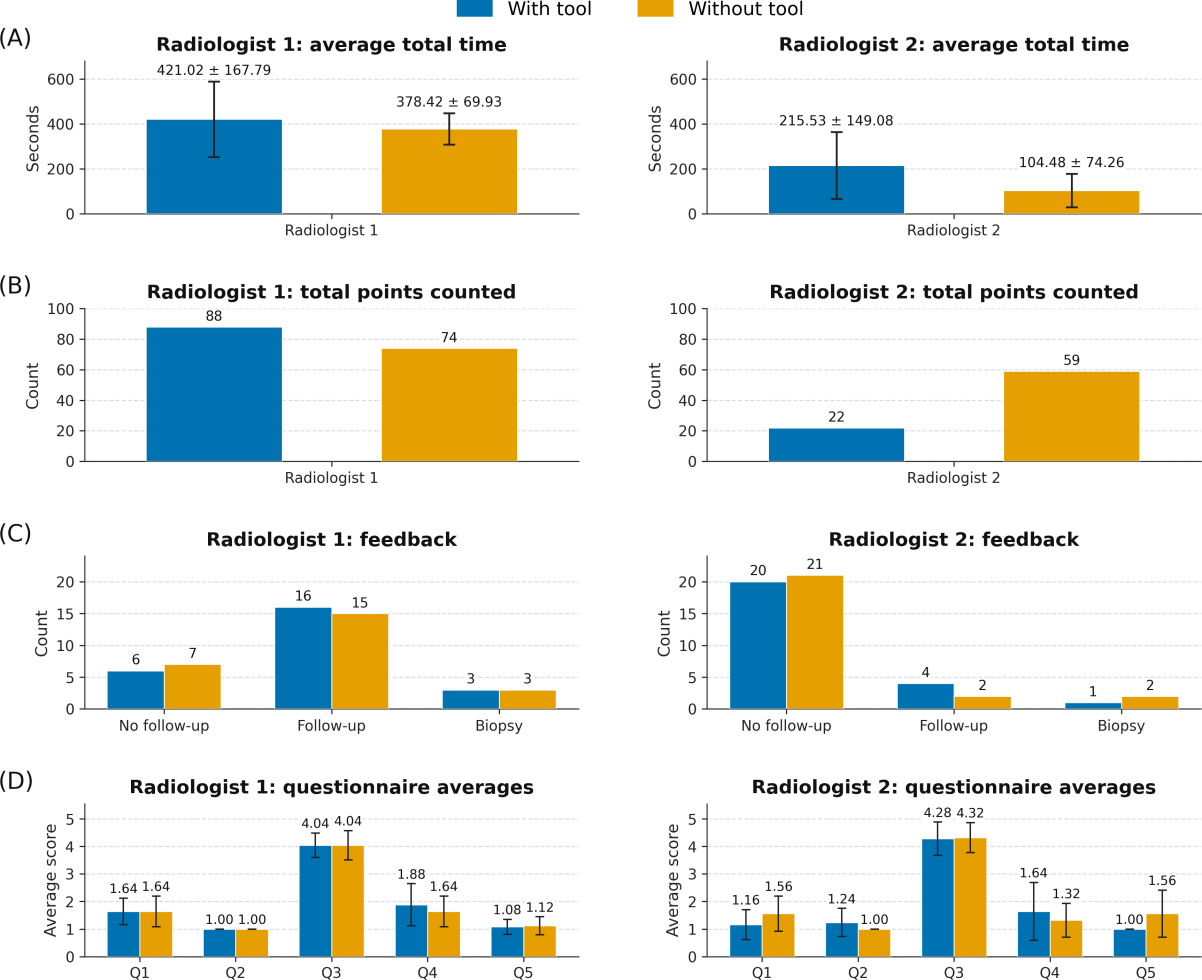
Reader-study results by radiologist and condition. (A) Mean evaluation time per volume; error bars indicate SD. (B) Total number of reader-marked candidate lesion locations across cases. (C) Counts of follow-up recommendations (no follow-up, follow-up, and biopsy). (D) Mean postcase Likert scores (1-5) for mental demand, hurriedness, perceived success, effort, and negative affect/stress; error bars indicate SD. “With tool” refers to cases read with the assistance of the AI overlay tool, and “Without tool” refers to cases read without it.

As shown in Figure 5, the average total time to evaluate each wbMRI for both radiologists was longer with the AI tool than without the AI tool. The use of the AI tool impacted the radiologists differently. While radiologist 1 spent more total time labeling the studies than radiologist 2, there was only a marginal time difference spent on labeling the studies with the AI tool than without the AI tool (Figure 5)—indicating that the AI tool only slightly increased the reporting times. Across both conditions, radiologist 1 marked twice as many candidate lesion locations as radiologist 2 (162 vs 81) and in the AI-assisted cases, radiologist 1 marked four times as many candidate lesion locations as radiologist 2 (22 vs 88) (Figure 5). In addition, radiologist 1 recommended follow-ups or biopsies more often than radiologist 2. The consideration of several more lesions as potentially worrisome is a rational explanation as to why radiologist 1 spent more time on labeling the studies than radiologist 2.

In contrast, although the AI-assisted samples had a higher mean reporting time for radiologist 2, it reduced the number of candidate lesion locations as potentially containing a tumor/lesion, in comparison to labeling without the AI tool. Given the low density of tumor slices in the volumes, the AI tool may have been helpful in reducing the number of false positives for radiologist 2 (Figure 5). Radiologist 2 also selected a smaller number of lesions for biopsy/follow-up than radiologist 1.

With regard to feedback on follow-up suggestions, the 2 radiologists overlapped 11 out of 25 (44.0%) times in their follow-up suggestions with the tool and 10 out of 25 (40.0%) times without the tool. With the tool, the 11 overlaps came from 6 overlaps in “No follow-up,” 4 overlaps in “Follow-up,” and 1 overlap in “Biopsy.” Without the tool, the 10 overlaps came from 7 overlaps in “No follow-up,” 2 overlaps in “Follow-up,” and 1 overlap in “Biopsy.”

Finally, the questionnaire was used to evaluate the subjective experience after each case. Reader 1 reported slightly higher effort with AI assistance (Q4) and slightly lower negative affect/stress with AI assistance (Q5). Reader 2 reported lower mental demand with AI assistance (Q1), higher hurriedness (Q2), similar perceived success (Q3), higher effort (Q4), and lower negative affect/stress (Q5). Because only 2 readers participated, these questionnaire findings should be interpreted descriptively rather than as generalized user-preference outcomes.

## Discussion

This study contributes both a pediatric surveillance wbMRI tumor-likelihood overlay and an exploratory reader-study evaluation of how such an overlay may affect radiologist workflow. In addition to machine learning metrics, other considerations such as deployment scenario, human-computer interaction, and individual differences must be taken into account.

The low signal-to-noise ratio of pediatric wbMRI scans and the limited number of positive cases contributed to labeling difficulties in this study. While the AI tool aimed to mitigate the challenges of tumor detection, radiologists still seemed to double-check the AI’s positive and negative suggestions, possibly subconsciously, in addition to accomplishing the required task. This increased the time and impacted the efficiency of their assessments in different ways based on the radiologist.

Model development occurred in an extremely low-prevalence setting. The small number of tumor-positive volumes limited the diversity of tumor appearances, sizes, and anatomic locations represented in training and likely contributed to unstable performance estimates and persistent false positives. In addition, the intentionally enriched patch-sampling strategy improved learnability but does not reproduce real-world prevalence. A limitation of this study is that the model showed modest lesion-level performance with a substantial false positive burden. Radiologist behavior may reflect responses to an occasionally imperfect or noisy overlay in addition to the effect of a clinically reliable tumor-detection tool. These factors limit robustness and generalizability, and external validation in larger, more diverse, preferably multicenter cohorts is needed.

In the United States, radiologists have faced a high number of lawsuits due to missed diagnoses [39]. Therefore, radiologists generally prefer to “over-call” a finding rather than “under-call” or miss out on potentially lethal findings. For a tool designed to detect tumors to be adopted, it is crucial to have a low false negative rate. However, this does not justify the tool having a very high false positive rate, which then introduces a new challenge of differentiating between the true positives and the false positives, which in itself may also have adverse legal ramifications.

The main purpose of surveillance wbMRI in patients with cancer predisposition disorders is to avoid missed tumors. However, false positives are not a trivial trade-off in this setting. If the tool highlights many nontumor regions, radiologists must spend additional time verifying them, clinician trust in the AI overlay may decline, and patients and families may be exposed to additional follow-up imaging, biopsy discussions, or uncertainty. In Li-Fraumeni surveillance, where families already face substantial psychological burden, false positive prompts may also increase anxiety if they trigger further workup for ultimately benign findings. Reducing false positives while preserving sensitivity is therefore central to clinical adoption.

Our results reflect the nuanced impact of AI tools on clinical practice, with both radiologists demonstrating markedly different responses to the tool’s guidance. The AI tool appeared to prolong the diagnostic process for one of the participating radiologists, possibly due to second-guessing the AI’s recommendations, overrelying on the recommendations, or trying to verify its findings. In contrast, the other radiologist seemed to use the tool to streamline their decision-making in the sense that although more time was spent with the AI tool, the number of candidate lesion locations selected was much lower with the tool than without the tool, suggesting that the AI tool was able to root out a lot of false-positive cases. The subjective survey responses also suggest that the same AI overlay can be experienced as either helpful or effortful, depending on the individual reader, reinforcing that user experience should be considered reader-specific rather than uniform.

The divergent behavior of the 2 readers suggests that identical AI overlays may not be interpreted consistently across radiologists. One reader marked substantially more candidate lesions and recommended follow-up more often overall, whereas the other was more conservative. This variability indicates that overlay interpretation is itself a human-factors problem: the same AI output may be integrated differently depending on reader thresholds, risk tolerance, and trust calibration. Future deployment studies should therefore include reader onboarding, calibration, and interface testing rather than assuming a one-size-fits-all interaction model.

While many AI tools often achieve diagnostic results that are comparable to radiologists, their narrow task focus and need for large, annotated datasets limit their effectiveness. Furthermore, the “black box” nature of neural networks means that radiologists often second-guess AI outputs. The differences between radiologists’ performance in this study suggest that some radiologists might spend extra time verifying AI outputs or modifying their usual workflow to incorporate AI feedback.

Our exploratory reader study reveals how individual radiologists’ diagnostic workflows change with the introduction of AI tools in varying ways and illustrates how individual biases and preferences affect human-AI collaboration. Another study suggests that self-confidence, not confidence in AI, affects the decision to accept or reject AI suggestions the most [40]. Given that only 2 readers were studied, these differences should be interpreted as preliminary evidence of reader-specific heterogeneity rather than as a comprehensive characterization of interreader variability in clinical practice.

Our analysis revealed that the AI tool impacted radiologists differently. Radiologist 1 displayed little difference in average evaluation time with or without the AI tool, while radiologist 2 had a lower mean evaluation time labeling without the tool than with it. The AI tool appeared to streamline radiologist 2’s decision-making process but also led to fewer candidates being selected, suggesting better exclusion of false positives. Despite the variance in time spent to conduct the requested tasks, the overlap in follow-up suggestions was similar for both radiologists with and without the AI tool in less than half the cases. More caution seems to be taken with the AI tool than without the AI tool with regard to the follow-up suggestions. Additionally, questionnaire results demonstrated varying subjective impacts on mental demand, hurriedness, irritation, workload, and perceived success with and without the tool.

Following the evaluation of our AI tool by 2 radiologists, we identified several challenges and opportunities in improving the usability of AI tools in health care. Based on the lessons learned from this study, the key insights are as follows:

- Different health care professionals with similar levels of training may interact with AI tools in various ways, influenced by personal biases and cognitive strategies, so calibrating AI tools to each individual represents a potentially promising future direction to explore.
- The opaque or black-box nature of many AI algorithms can lead to distrust and second-guessing of AI outputs by health care professionals; therefore, improved explainability and interpretability may mitigate clinician skepticism and reduce redundant verification behaviors.
- Having clear and comprehensive training for health care professionals on how to effectively use AI tools is important in ensuring the proper use of the tools and improving satisfaction with their utilization.
- Poor integration of AI tools into existing workflows can disrupt routine practices and reduce overall efficiency, so employing a continuous evaluation and improvement process to refine AI tools based on real-world performance and user feedback may prove useful.
- The introduction of AI tools can increase cognitive load and mental demand if the interactive component is not up to par. Therefore, making health care professionals a part of the discussion on how to develop user-friendly AI tools that can help reduce friction with existing workflows is crucial.
- The technical complexity of AI tools can pose a barrier to their effective use by health care professionals who are not technically inclined. Therefore, it is important to create user-centric designs that serve the needs and preferences of health care professionals from a wide spectrum of experiences.
- Fostering collaboration between AI developers, health care professionals, and researchers in the development process to ensure AI tools are clinically relevant and serve precise clinical needs.
- Encouraging the use of AI tools as supportive aids, rather than replacements for human imaging interpretation, can help avoid overconfidence or underconfidence in the AI tools.

In summary, this study addresses a clinically specific and technically challenging setting, pediatric surveillance wbMRI in LFS, and shows that AI overlays can influence radiologist workflow in reader-specific ways. We did not formally disentangle appearance-driven signals from implicit contextual/location priors, so future work should test whether the model remains sensitive to tumors in less commonly represented locations. The reader study should be interpreted as preliminary. Only 2 radiologists participated, and the design was not randomized or counterbalanced. Therefore, the observed differences in timing, candidate-lesion marking, and subjective responses should be viewed as descriptive and hypothesis-generating rather than definitive estimates of clinical workflow impact or trust or as robust estimates of population-level reader effects. The nuanced relationship between radiologists and AI tools requires further research to optimize these systems for diverse diagnostic workflows. This study emphasizes the interplay between trust, validation, and interpretation in human-AI collaboration, highlighting that a one-size-fits-all strategy may be inadequate for optimizing AI tools in health care.

## Abbreviations

LFS: Li-Fraumeni syndrome
MRI: magnetic resonance imaging
STIR: Short Tau Inversion Recovery
wbMRI: whole-body magnetic resonance imaging
